# Socioeconomic and sex inequalities in parent‐reported adolescent mental ill‐health: time trends in four British birth cohorts

**DOI:** 10.1111/jcpp.13730

**Published:** 2022-12-20

**Authors:** Eoin McElroy, Marc Tibber, Pasco Fearon, Praveetha Patalay, George B. Ploubidis

**Affiliations:** ^1^ School of Psychology Ulster University Coleraine UK; ^2^ Centre for Longitudinal Studies, UCL Social Research Institute University College London London UK; ^3^ Research Department of Clinical, Educational and Health Psychology University College London London UK; ^4^ MRC Unit for Lifelong Health and Ageing University College London London UK

**Keywords:** Socioeconomic inequalities, sex inequalities, emotional problems, behavioural problems, time trends, ALSPAC

## Abstract

**Background:**

Studies using symptom‐based screeners have suggested that mental ill‐health has increased in adolescents in recent decades, however, few studies have tested the equivalence of their instruments, which is critical for inferring changes in prevalence. In addition, little research has explored whether socioeconomic position (SEP) and sex inequalities in adolescent mental health have changed over time.

**Methods:**

Using structural equation modelling, we explored SEP and sex differences in harmonised parent reports of emotional and behavioural problems, using data from four UK birth cohorts: the 1958 National Child Development Study (NCDS'58; *n* = 10,868), the 1970 British Cohort Study (BCS'70; *n* = 8,242), the 1991–92 Avon Longitudinal Study of Parents and Children (ALSPAC'91; *n* = 5,389), and the 2000–01 Millennium Cohort Study (MCS'01; *n* = 9,338).

**Results:**

Compared with the two earliest cohorts, members of MCS'01 had higher latent mean scores on emotional problems (both sexes), and lower scores on behavioural problems (females only). The associations between four indicators of SEP and emotional problems were strongest in MCS'01, with housing tenure having the strongest association. All four SEP indicators were associated with behavioural problems in each cohort, with housing tenure again more strongly associated with problems in the MCS'01. Mediation analyses suggested that the increase in emotional problems occurred despite broadly improving socioeconomic conditions.

**Conclusions:**

Our findings suggest that parent reports of adolescent emotional problems, but not behavioural problems, have risen in recent generations and this trend is not solely due to reporting styles. A failure to address widening social inequalities may result in further increases in mental ill‐health amongst disadvantaged young people.

## Introduction

Mental health problems often emerge in adolescence (Wagner et al., 2017), show strong levels of persistence into adulthood (Ormel et al., 2015), and are associated with a range of negative personal and economic outcomes later in life (Copeland, Wolke, Shanahan, & Costello, 2015; Ploubidis, Batty, Patalay, Bann, & Goodman, 2021). Studies of secular/time trends in adolescent mental health are, therefore, important, as they can influence policy (Abdallah, 2018), and can also help us gauge the success of public health interventions aimed at reducing the burden of mental ill‐health (Collishaw, 2015).

Studies using parent reports have consistently observed increases in both emotional and behavioural problems in more recent cohorts (Coley, O'Brien, & Spielvogel, 2019; Högberg, Strandh, & Hagquist, 2020; Patalay & Gage, 2019; Pitchforth et al., 2019; van Vuuren, Uitenbroek, van der Wal, & Chinapaw, 2018). However, to the best of our knowledge, none of these studies has explicitly tested the measurement equivalence of their instruments across cohorts. Even when the same measures are administered across studies, differences in administration (e.g. pen‐and‐paper vs. computer‐assisted; Reichmann et al., 2010) and other methodological factors (e.g. order of questionnaires; Bowling, 2005) may introduce systematic biases into the data. Moreover, responses to questions may systematically vary across cohorts due to changes in social and cultural norms (Ploubidis, McElroy, & Moreira, 2019). For instance, increased awareness of mental health, and reduced stigma associated with help‐seeking, may have increased the level of reporting of emotional and behavioural problems in more recent generations. As such, the first aim of this study is to use a latent variable modelling approach to explore differences in latent means across four British birth cohorts (children born 1958–2002), thus allowing us to directly address measurement invariance.

Beyond simply charting prevalence rates, cross‐cohort studies may also help explain why adolescent mental health problems have fluctuated over time, which again may be used to drive interventions and policy (Collishaw, 2015). Low socioeconomic position (SEP) is robustly associated with an increased risk of emotional and behavioural problems in young people (Langton, Collishaw, Goodman, Pickles, & Maughan, 2011; Reiss, 2013). However, despite widening socioeconomic inequalities, few studies have explored whether changes in national socioeconomic contexts have been associated with a rise or fall in adolescent mental health (van Vuuren et al., 2018). Collishaw, Furzer, Thapar, and Sellers (2019) explored whether the associations between parental income and parent‐ and teacher‐reported mental health problems changed across three cohorts of 11‐year‐olds (assessed in 1999, 2004, and 2012). Their analyses compared total scores on the Strengths and Difficulties Questionnaire (SDQ; Goodman, 1997) between families that were below or above the bottom quintile of incomes within each cohort. They found that, for parent‐reported problems only, the disparity between the two groups widened between 1999 and 2012. However, further research is required to determine whether this disparity has increased over a longer period, and whether this increase applies to more specific forms of mental health ill‐health (e.g. emotional vs. behavioural problems). Furthermore, SEP is a multifaceted construct comprised of interrelated factors, and recent research suggests that parental income is a weaker predictor of adolescent emotional problems than other indicators of SEP (Langton et al., 2011; Moulton, Goodman, Nasim, Ploubidis, & Gambaro, 2021). As such, there is a need for additional research looking at the potential widening of mental health inequalities across various socioeconomic markers.

The scarcity of research in this area may be due to a lack of datasets from different decades that have comparable indicators of childhood SEP and mental health outcomes. Retrospective harmonisation (i.e. the derivation of comparable variables) offers a means of comparing trends across studies that lack common instruments (McElroy et al., 2021). To our knowledge, only one study has used this approach to explore SEP inequalities in adolescent mental health – Langton et al. (2011) found evidence of an increased income differential in the emotional problems of 15‐/16‐year‐olds assessed between 1974 and 2004.

We aim to expand upon this by exploring SEP inequalities in adolescent emotional and behavioural problems over an even broader period of time (1974–2018) and to formally test whether changes in SEP can explain (i.e. mediate) any cross‐cohort differences in prevalence that are observed.

In addition, few studies have explored whether the well‐documented sex differences in adolescent emotional and behavioural problems (Högberg et al., 2020; Pitchforth et al., 2019; van Vuuren et al., 2018) have widened or narrowed over time. Therefore, our final aim is to look at cross‐cohort sex differences in emotional and behavioural problems, using an appropriate latent variable modelling approach.

## Methods

### Data

We used data from four British birth cohort studies. The National Child Development Study (NCDS'58) follows the lives of 17,415 people that were born in England, Scotland, or Wales in a single week in March 1958 (Power & Elliott, 2006). Similarly, the British Cohort Study (BCS'70) tracks 17,198 people born in England, Scotland, and Wales in a single week in March 1970 (Elliott & Shepherd, 2006). The Avon Longitudinal Study of Parents and Children (ALSPAC'91) is a regional birth cohort that tracks the lives of people born in the former county of Avon between April 1991 and December 1992 (Boyd et al., 2013). The initial number of pregnancies enrolled is 14,541. Of these initial pregnancies, there was a total of 14,676 foetuses, resulting in 14,062 live births and 13,988 children who were alive at 1 year of age (Boyd et al., 2013). The Millennium Cohort Study (MCS'01) is a UK‐wide birth cohort study of individuals born at the start of the millennium (September 2000–January 2002), with the initial sample consisting of 19,517 children (Connelly & Platt, 2014). Ethics, data access, acknowledgements, and funding statements are provided in the Supporting Information (Appendix S1), and further information on these cohorts is available elsewhere (CLOSER, 2021). Our analytic samples consisted of singletons and first‐born twins who had any data on our outcome variables (mental health at age 16/17), and had data on at least one indicator of SEP up to age 11: *N*_NCDS'58_ = 10,868; *N*_BCS'70_ = 8,242; *N*_ALSPAC'91_ = 5,389; *N*_MCS'01_ = 9,338.

### Measures

#### Adolescent mental health

In both the NCDS'58 and BCS'70, mental health was assessed using parent‐report versions of the Rutter Behaviour Scales (Rutter, Tizard, & Whitmore, 1970) when children were aged 16. Items covered a range of emotional and behavioural problems, with responses indicated on a 3‐point Likert response scale (0 = ‘Does not apply’, 1 = ‘Applies somewhat’, 2 = ‘Certainly applies’). In both the ALSPAC'91 (at age 16) and MCS'01 (at age 17), parent‐report versions of the SDQ (Goodman, 1997) were completed. This scale consists of 25 items (3‐point Likert; 0 = ‘Not true’, 1 = ‘Somewhat true’, 2 = ‘Certainly true’) that assess five domains: emotional problems, peer problems, behavioural problems, hyperactivity, and prosocial behaviour.

As the Rutter scale is a precursor to the SDQ, the two scales share similar items and a comparable Likert scoring system. We, therefore, derived harmonised scales of emotional and behavioural problems based on conceptually similar items from across the two measures using a content validation approach. Candidate items for harmonisation were identified by two independent raters (EM and MT). Both raters scrutinised every available question within each measure and assigned each item a code reflecting its core content. Initial inter‐rater agreement was high (88%). In instances where the two raters disagreed on the coding of an item, a third independent rater (PF) made the decision on which item code (if either) was appropriate. This process led to a final harmonised subset consisting of three questions tapping emotional problems, and four questions capturing behavioural problems (Table S1).

#### Socioeconomic position

Four harmonised indicators of SEP were used in our analyses.

##### Paternal occupation

We used previously derived harmonised measures of paternal occupational status when children were aged 11 (aged 10 in BCS'70; Dodgeon et al., 2018). These variables were based on the study fathers' occupational status as classified under the 1990 Registrar General's Social Class system. Further details of these measures are available elsewhere (Dodgeon et al., 2018). Binary variables were derived from these categories (0 = non‐manual; 1 = manual occupation).

##### Maternal and paternal education

Separate paternal and maternal education variables were included that reflected whether cohort mothers and fathers completed any post‐compulsory education (0 = remained after the compulsory period; 1 = left school at minimum age).

##### Housing tenure

Binary variables were derived based on the type of accommodation in which study children lived in early‐to‐mid childhood. Information on housing tenure was available in NCDS'58 at age 7, BCS'70 at age 5, ALSPAC'91 between pregnancy and 12 months post birth, and MCS'01 at age 5. Harmonised variables were created and scored as 0 (lives in a house that is owned/mortgaged) and 1 (other, e.g. private rental, social housing).

Rates of missingness for all study variables are presented separately by cohort in Table S2.

### Analysis

To ensure that cross‐cohort comparisons of the means and covariances of the harmonised mental health variables were valid, we tested for measurement invariance using multigroup confirmatory factor analysis (CFA). Details of this procedure are available in the Supporting Information (Appendix S2).

After establishing a sufficient level of measurement invariance, we tested for differences in latent means across the cohorts using a structural equation modelling (SEM) framework. This approach had the advantage of allowing us to compare emotional and behavioural factors across cohorts while accounting for measurement error (Bollen, 2014). Following standard practice (Aiken, Stein, & Bentler, 1994), latent means were fixed to zero in a reference group, NCDS'58, and freely estimated in all other groups using the best‐fitting measurement invariance model from the previous step. The estimated latent means in each of the non‐reference cohorts represented the latent mean differences between the relevant cohort and NCDS'58. In order to explore the potential impact of differential sample attrition, estimates of latent means were also produced using multiple imputations, and these values were compared with the estimates produced using the originally defined complete‐case analytic samples (see Appendix S3 for a description of the imputation strategy). The MCS'01 is the only cohort in the present study that incorporates design features such as clustering, stratification, and weighting. We incorporated these in the analysis of the MCS'01 cohort, but in order to be consistent with the other cohorts, we included design weights only and did not use non‐response weights, as missing data due to non‐response were handled with multiple imputations. Given that the outcome measures were collected slightly later in the MCS'01 (age 16/17) compared with the other cohorts (age 16), we conducted a sensitivity analysis in which the latent means of emotional and behavioural problems from the prior sweep of the MCS (age 14/15) were compared with the age 16 data from the other cohorts.

Next, we examined potential cross‐cohort differences in the associations between our SEP indicators and adolescent mental health, first by testing cohort × SEP interactions, and then by conducting cohort‐stratified analyses. We used multiple causes multiple indicators (MIMIC) models, in which correlated latent emotional and behavioural variables were regressed on our indicators of SEP and sex (Figure S1). The MIMIC models were estimated in Mplus version 8.3 (Muthén & Muthén, 2017) using robust maximum likelihood (MLR), which deals with missing data under the missing at random (MAR) assumption. Again, sensitivity checks with emotional and behavioural problems at age 14/15 in MCS'01 as outcomes were conducted.

#### Mediation

To analyse whether changes in SEP could explain any cross‐cohort differences in adolescent mental health, we used the potential outcomes approach to test for mediation (De Stavola, Daniel, Ploubidis, & Micali, 2015). This approach divides the total causal effect of the exposure on the outcome into two distinct effects: the total natural indirect effect (TNIE) and the pure natural direct effect (PNDE). The TNIE represents the effect that operates through the mediator variable, whereas the PNDE captures all pathways other than through the mediator. This method has the advantage of accounting for exposure‐mediator interactions, a source of potential bias in traditional approaches to mediation (VanderWeele & Vansteelandt, 2014). For our analyses, our exposure variables were dummy variables reflecting cohort membership (NCDS'58 used as reference category), and our outcomes were latent emotional and behavioural variables. Separate models were estimated with each of our indicators of SEP acting as mediators and a model with all four indicators of SEP included was estimated as a sensitivity check for intermediate confounding.

## Results

**Figure 1:**
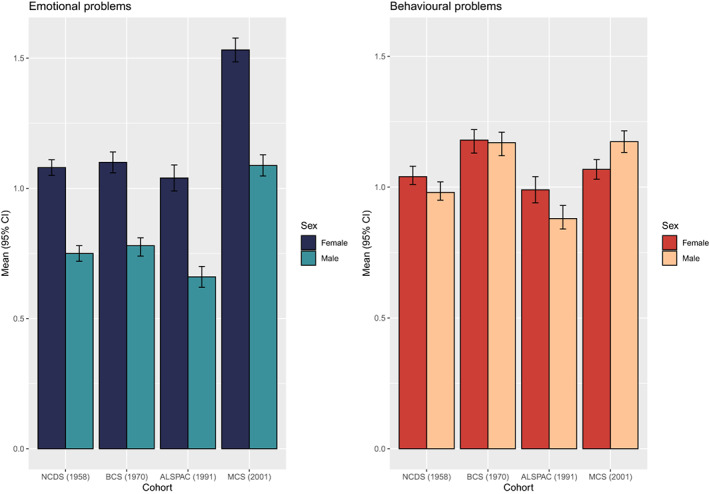
Means and 95% confidence intervals of harmonised emotional and behavioural problem scales across cohorts and gender

Descriptive statistics for our harmonised measures of mental health are presented in Figure 1, and the distributions of these scores are presented in the Supporting Information (Figures S1 and S2). Based on the observed scores, emotional problems were highest in MCS'01. Behavioural problems were comparably high in BCS'70 and MCS'01 for males only. Within each cohort, females consistently scored higher than males on emotional problems, whereas sex differences in behavioural problems were less pronounced. Frequencies and percentages of participants in the different SEP categories are available in the Supporting Information (Table S3) and show an increase in the proportion of participants in the higher SEP groups in more recent cohorts.

### Time trends in adolescent mental health

The results from our tests of measurement invariance across cohorts are presented in Table S4, and distributions of scores on the latent variables are shown in Figure S3. The configural model demonstrated acceptable levels of model fit in both the overall and sex‐stratified samples (factor loadings and thresholds from the configural model are shown in Figures S4 and S5). Correlations between the latent emotional and behavioural factors ranged from 0.57 (NCDS'58) to 0.64 (BCS'70). The scalar model, however, resulted in a worsening of fit according to conventional model comparison guidelines (∆CFI > .01). An inspection of the modification indices suggested fit could be improved by freeing the threshold parameters of three indicators: ‘disobedience’, ‘aggression’, and ‘fear/anxiety’. This partial scalar model resulted in permissible reductions in fit (∆CFI < .01; ∆RMSEA < .01), and given that the majority of indicators remained invariant, valid comparisons of mean scores could be made across cohorts (Little, 2013).

Results from these latent mean comparisons are shown in Table S5. Treating the earliest cohort (NCDS'58) as the reference group, we found that emotional problems were significantly higher for both males and females in the MCS'01, and lower in the ALSPAC'91 cohort for males only. Behavioural problems were lower for both sexes in ALSPAC'91, were lower for females in MCS'01, but were higher for males and females in BCS'70. Both fit statistics and estimates of mean differences were consistent across the non‐imputed and imputed samples (Tables S4 and S5). The results from the sensitivity check (outcomes from age 14/15 sweep in MCS'01) also showed increases in emotional problems in MCS'01 compared with earlier cohorts, suggesting the increase is not due to heterogeneity in the age of assessment. However, this analysis found increases in behavioural problems in both sexes in MCS'01, which may be due to the younger age of assessment.

### Socioeconomic inequalities in adolescent mental health across cohorts

**Figure 2:**
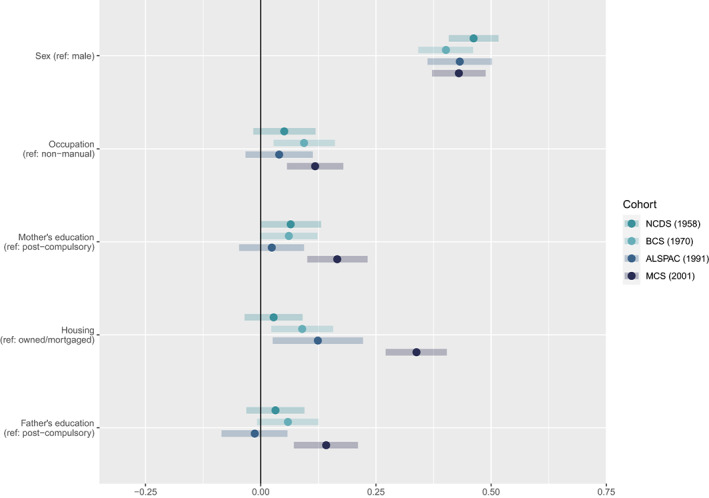
Standardised regression coefficients and 95% confidence intervals showing associations between socioeconomic position (SEP) indicators (Y‐axis) and adolescent emotional problems (X‐axis) by cohort. Reference categories for SEP predictors shown in brackets

**Figure 3:**
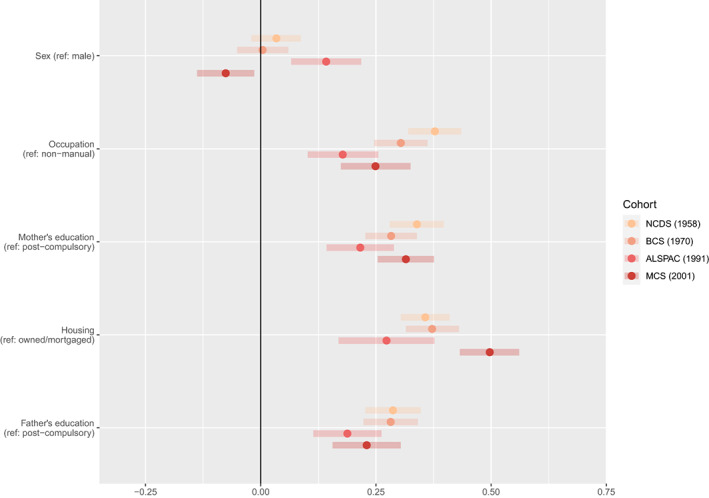
Standardised regression coefficients and 95% confidence intervals showing associations between socioeconomic position (SEP) indicators (Y‐axis) and adolescent behavioural problems (X‐axis) by cohort. Reference categories for SEP predictors shown in brackets

Across all four indicators of childhood SEP, we found evidence of cohort x SEP interactions, supporting our presentation of cohort‐stratified analyses. Standardised regression coefficients and 95% CIs from our MIMIC models are presented in Figures 2 and 3 (see Figure S6 for age 14/15 sensitivity analysis). All four indicators of socioeconomic disadvantage were associated with emotional problems in MCS'01, with housing tenure showing the largest effect size. In the three earlier cohorts, only housing tenure (in ALSPAC'91 and BCS'70) and the father's occupation (in BCS'70) were associated with adolescent emotional problems. A lack of overlap in 95% CIs suggested that the association between housing tenure and adolescent emotional problems was stronger in MCS'01 compared with earlier cohorts. All four indicators of disadvantage were associated with greater behavioural problems in all of the cohorts, however, the association with housing tenure was stronger in MCS'01.

The results from the mediation analyses are presented in Table S6. Across all four of the mediators (indicators of SEP) the TNIEs were generally small, were positive for BCS'70, and were negative for ALSPAC'91 and MCS'01. For emotional problems, the largest TNIE was observed when housing tenure was treated as the mediator in MCS'01 (*b* [95% CI] = −0.064 [−0.076; −0.051]). For behavioural problems, maternal education in MCS'01 produced the largest TNIE (*b* [95% CI] = −0.081 [−0.099; −0.064]). The negative TNIEs reflect inconsistent mediation or regression suppression (Friedman & Wall, 2005; MacKinnon, Krull, & Lockwood, 2000). Inconsistent mediation occurs when the direct and indirect effects from the exposure to the outcome have different signs. In other words, rather than explain the observed difference between cohorts, the SEP variables may inflate the observed differences when they are included in the model, or ‘suppress’ them when they are excluded. This was supported by comparing regression models with and without indicators of SEP included as confounders (Table S7). In the models in which the SEP indicators were included, the direct effects of the cohort dummies on mental health outcomes were stronger. The negative direction of the NIEs was due to the negative association between the cohort dummy indicators and the SEP mediators, which were coded to indicate disadvantaged SEP. This pattern of results can be interpreted as follows – a greater proportion of participants were in more favourable SEP categories in the more recent cohorts, however, despite this, levels of mental health problems were higher in these cohorts.

### Sex inequalities

Looking at sex differences (Figures 2 and 3), females had greater emotional problems than males and the magnitude of this difference did not change across cohorts. Females had fewer behavioural problems in MCS'01, and, contrary to established trends, greater levels of behavioural problems in ALSPAC'91.

## Discussion

The present study explored differences in parent‐reported emotional and behavioural problems in adolescence across four British birth cohorts, spanning a period of approximately 42 years. We found evidence of an increase in emotional problems for both males and females in the most recent cohort. Trends in behavioural problems were more variable – levels increased between NCDS'58 and BCS'70 but had decreased by MCS'01. Indeed, behavioural problems were lower for females in MCS'01 compared with NCDS'58. By establishing partial measurement invariance, we can conclude that the observed differences in prevalences were not solely an artefact of systematic differences in measurement error across the cohorts. In other words, cohort parents interpreted the questions in a broadly similar manner across generations. This extends existing research (Coley et al., 2019; Högberg et al., 2020; Patalay & Gage, 2019; Pitchforth et al., 2019; van Vuuren et al., 2018) by confirming that the increase in adolescent emotional problems observed in recent years is not entirely the result of changes to societal/cultural attitudes, such as increased mental health awareness, lower levels of stigma, and a greater willingness to report symptoms.

Our results suggested that members of the ALSPAC'91 cohort had lower levels of emotional and behavioural problems than those in the other cohorts. However, this may be explained, at least in part, by the fact that ALSPAC'91 is not a nationally representative cohort, with participants coming from a relatively affluent region in the west of England (Boyd et al., 2013). As such, from the current data, it was not possible to determine whether the increase in emotional problems observed between NCDS'58 and MCS'01 reflected a gradual or non‐linear increase at the national level, or whether the rise observed in the MCS'01 was cohort‐specific. Data from other representative cohorts (either ongoing or future cohorts) will be required to shed light on these trends.

Our second aim was to explore whether changes in SEP could account for these cross‐cohort changes in mental ill‐health in young people. The associations between SEP indicators and emotional problems were strongest in MCS'01, with the association between housing tenure and emotional problems particularly strong in this cohort. There were no clear cross‐cohort differences in the strengths of associations between SEP indicators and behavioural problems, with the exception of housing tenure which again was more strongly associated with difficulties in the MCS'01.

There are several explanations as to why housing tenure is so strongly associated with adolescent mental health. A recent review argued that, although housing is a key factor in the emergence and maintenance of health inequalities, the pathways between these domains are complex and multifaceted (Swope & Hernández, 2019). Home ownership can act as a proxy for general financial security, which has a number of direct and indirect benefits for mental health. Examples include reduced financial worry (direct; Netemeyer, Warmath, Fernandes, & Lynch, 2018) and healthier behaviours such as diet and exercise (indirect; Conry et al., 2011). Home ownership is associated with better housing quality, and lower levels of over‐crowding, both of which have an association with mental health (Swope & Hernández, 2019). It is also associated with parental mental health health and family conflict (Cairney & Boyle, 2004), which are key drivers of mental ill‐health in young people. Furthermore, the neighbourhood environment may be more conducive to positive mental health in affluent, high‐ownership areas (e.g. better access to amenities, lower levels of antisocial behaviour; McElroy et al., 2021).

As for the increase in the association between housing and adolescent emotional and behavioural problems, this trend could be explained by changes to UK housing policy over this period. Prior to the 1970s, affordable public housing in the United Kingdom was available to individuals and families from a range of socioeconomic backgrounds. Over the past 50 years, however, social housing has gone through a process of ‘residualisation’ (Pearce & Vine, 2014), wherein it has become more of a safety net for individuals unable to obtain accommodation in the private sector (e.g. due to poverty or health reasons). As such, adolescents living in non‐owned houses in more recent cohorts may be disadvantaged in other domains to a greater degree than in previous generations (Langton et al., 2011). Compounding this, members of the MCS'01 transitioned from childhood through to adolescence during a period of considerable austerity, with cuts to public spending having the most pronounced impact on those with low income (Barr, Kinderman, & Whitehead, 2015). Therefore, this change in the make‐up of the population who do not own their own house, and the widening socioeconomic inequalities more broadly, may account for this increased association between housing tenure and adolescent mental health in the more recent cohorts.

Our formal tests of mediation were supportive of the above, suggesting changes in socioeconomic inequalities have had an impact on the mental health of UK adolescents. We found evidence of ‘inconsistent mediation’ or ‘regression suppression’ in both ALSPAC'91 and MCS'01, suggesting that the exclusion of SEP indicators serves to reduce or suppress the cross‐cohort differences. Although this pattern does not explain the observed differences between the cohorts, it does offer a relatively straightforward interpretation. First, there was an overall improvement in social and economic conditions in the United Kingdom between 1958 and 2000, such as a greater proportion of parents owning/mortgaging their homes (Pearce & Vine, 2014). However, despite these overall improvements in conditions, adolescent mental health problems increased in the population over the same period. The increased associations between disadvantaged SEP and emotional and behavioural problems in the most recent cohort suggest that economic disadvantage represents a greater risk factor for mental health problems in more recent cohorts. This trend could be seen as consistent with widening social inequalities in the United Kingdom, with those at the lower end of the distribution facing ever more challenges across multiple domains (Pickett & Wilkinson, 2010).

These findings have implications for social and public health policy, as a failure to address widening social inequalities may see further increases in adolescent mental health problems in young people who face the most adverse social conditions (Tibber, Walji, Kirkbride, & Huddy, 2021). Strategies to mitigate this trend are even more important given that recent evidence suggests that the COVID‐19 pandemic may have widened inequalities in the United Kingdom even further (Blundell, Costa Dias, Joyce, & Xu, 2020).

Consistent with a wide body of evidence, emotional problems were more common in females than males in all four cohorts, whereas sex differences in behavioural problems were less consistent (Pitchforth et al., 2019; van Vuuren et al., 2018). Behavioural problems were slightly less common in females in the MCS'01, and in ALSPAC'91 females had greater levels of behavioural problems than males. The reasons for these discrepancies remain unclear. One factor may be the reliance on parent reports in the present study – some have suggested that teacher reports better capture behavioural problems as these may be more salient in classroom environments (Humphrey & Wigelsworth, 2016). Further cross‐cohort research into sex inequalities in behavioural problems, using different informants is, therefore, needed. The lack of cross‐cohort change in emotional problems suggests that further efforts are required to reduce this mental health gap between male and female adolescents.

Given that the results of our mediation analyses do not account for the overall increase in emotional symptoms across cohorts, the reason for this rise remains unclear – as does the fact that the rise was seen in emotional problems but not behavioural problems. There are various factors that could account for this change that could be explored in future cross‐cohort studies. For instance, research has identified changes in various health‐related behaviours across earlier and more recent cohorts, such as increased sleep problems, and higher perceived weight and body mass index (BMI; Patalay & Gage, 2019), and there is some indication that the association of certain health factors (e.g. smoking, BMI) with emotional symptoms is stronger in more recent cohorts (Gage & Patalay, 2021). However, future work is needed to establish the direction of causality as negative health behaviours can also be an outcome of psychological distress, as well as the extent to which changes in negative health behaviours account for cross‐cohort differences. Other societal changes that might also contribute to the observed increases include technological advances in communication (Keles, McCrae, & Grealish, 2020), increased academic pressures (Curran & Hill, 2019), and intergenerational inequality (Johnston, Schurer, & Shields, 2013) amongst others. Understanding the drivers of these generational trends is necessary and there is little current evidence to explain the causes of these observed increases in emotional symptoms.

### Strengths and limitations

Strengths of this study include the use of four longitudinal UK population cohorts, three of which are nationally representative, and had comparable measures at similar time points. Our modelling approached the established partial measurement invariance, which allowed us to explore time trends across the cohorts while accounting for differences in measurement error. In terms of limitations, we relied on parent reports of emotional and behavioural problems, with research suggesting that parents may under‐report symptoms compared with adolescent self‐reports (Sourander, Helstelä, & Helenius, 1999). Furthermore, research suggests self‐reported mental health problems are more weakly associated with SEP compared with parent or teacher reports (Johnston, Propper, Pudney, & Shields, 2014). As such, further research using self‐report data is warranted to substantiate these widening inequalities. As is common practice, we relied on differences in practical fit indices to judge the level of measurement invariance across cohorts, however, this approach has not been systematically studied in the context of CFA models with ordered‐categorical indicators. Finally, our samples were drawn entirely from the UK population; while research supports the rise of parent‐reported emotional and behavioural problems in many high‐income countries, these findings may not generalise to low‐income countries and to self‐reports where there is greater inconsistency in trends in behavioural problems (Collishaw, 2015).

## Conclusions

The present study supports previous accounts that parent‐reported emotional problems have risen in more recent cohorts and that this increase is not solely an artefact of reporting practices. In addition, this rise occurred in spite of general improvements to socioeconomic conditions. In contrast, females born at the turn of the millennium had lower levels of behavioural problems compared with earlier cohorts. We also found that low SEP, particularly related to housing tenure, became more strongly associated with emotional and behavioural problems over the same period. Sex differences in emotional problems remained consistent over time, whereas females in the most recent cohort had fewer behavioural problems than males. Further work is required to understand the reasons behind the increase in adolescent emotional problems in more recent cohorts. A failure to address SEP inequalities may result in further increases in mental health problems amongst disadvantaged young people.

## Supporting information


**Appendix S1.** Ethics, data access, acknowledgements, and funding statements.
**Appendix S2.** Measurement invariance using multigroup confirmatory factor analysis.
**Appendix S3.** Multiple imputation strategy.
**Figure S1.** Diagram of MIMIC models.
**Figure S2.** Distributions of raw score of harmonised emotional and behavioural problem scales (by sex and total sample).
**Figure S3.** Latent score distributions of harmonised emotional and behavioural problem scales.
**Figure S4.** Standardised factor loadings from configural model (analytic samples).
**Figure S5.** Standardised thresholds from configural model (analytic samples). Thresholds are on a probit scale.
**Figure S6.** Sensitivity analysis – cohort stratified analyses with MCS emotional (left) and behavioural (right) problems at age 14/15.

**Table S1.** Harmonised emotional and behavioural problems sub‐scales.
**Table S2.** Missing data patterns.
**Table S3.** Frequencies and percent of participants by SEP indicators.
**Table S4.** Fit statistics from multi‐group CFAs/Table S4b. Modification indices.
**Table S5.** Comparison of latent means across cohorts.
**Table S6.** Results from mediation analyses.
**Table S7.** Sensitivity check. Models with and without SEP (housing) included as covariate.
