# Supplementary material for: Socioeconomic and sex inequalities in parent‐reported adolescent mental ill‐health: time trends in four British birth cohorts
Source: J Child Psychol Psychiatry. 2022 Dec 20;64(5):758–67. doi: 10.1111/jcpp.13730 (PMC10952603; doi:10.1111/jcpp.13730)
Supplement: Supplementary file 1 — Appendix S1. Ethics, data access, acknowledgements, and funding statements. Appendix S2. Measurement invariance using multigroup confirmatory factor analysis. Appendix S3. Multiple imputation strategy. Figure S1. Diagram of MIMIC models. Figure S2. Distributions of raw score of harmonised emotional and behavioural problem scales (by sex and total sample). Figure S3. Latent score distributions of harmonised emotional and behavioural problem scales. Figure S4. Standardised factor loadings from configural model (analytic samples). Figure S5. Standardised thresholds from configural model (analytic samples). Thresholds are on a probit scale. Figure S6. Sensitivity analysis – cohort stratified analyses with MCS emotional (left) and behavioural (right) problems at age 14/15. [file JCPP-64-758-s002.docx]

**Socioeconomic and sex inequalities in parent-reported adolescent mental ill-health: Time trends in four British birth cohorts**

**Supporting Information**

Contents

[Appendix S1. – Ethics, data access, acknowledgements, and funding statements 2](#_Toc119072642)

[Appendix S2. 3](#_Toc119072643)

[Appendix S3. 4](#_Toc119072644)

[Figure S1. Diagram of MIMIC models. 5](#_Toc119072645)

[Figure S2. Distributions of raw score of harmonised emotional and behavioural problem scales (by sex and total sample). 6](#_Toc119072646)

[Figure S3. Latent score distributions of harmonised emotional and behavioural problem scales. 7](#_Toc119072647)

[Figure S4. Standardised factor loadings from configural model (analytic samples). 8](#_Toc119072648)

[Figure S5. Standardised thresholds from configural model (analytic samples). Thresholds are on probit scale. 9](#_Toc119072649)

[Figure S6. Sensitivity analysis – cohort stratified analyses with MCS emotional (left) and behavioural (right) problems at age 14/15. 10](#_Toc119072650)

[References 11](#_Toc119072651)

### Appendix S1. – Ethics, data access, acknowledgements, and funding statements

*Ethics and data access statements*

The most recent sweeps of the **NCDS, BCS70**, and **MCS** have all been granted ethical approval by the National Health Service (NHS) Research Ethics Committee and all participants have given informed consent. Data for NCDS (SN 6137), BCS70 (SN 8547), MCS (SN 8682) and all four COVID-19 surveys (SN 8658) are available through the UK Data Service.

**ALSPAC:** Ethical approval for the study was obtained from the ALSPAC Ethics and Law Committee and the Local Research Ethics Committees. Informed consent for the use of data collected via questionnaires and clinics was obtained from participants following the recommendations of the ALSPAC Ethics and Law Committee at the time. Please note that the ALSPAC website contains details of all the data that is available through a fully searchable data dictionary and variable search tool (<http://www.bristol.ac.uk/alspac/researchers/our-data/>).

*Acknowledgements*

**ALSPAC:** We are extremely grateful to all the families who took part in this study, the midwives for their help in recruiting them, and the whole ALSPAC team, which includes interviewers, computer and laboratory technicians, clerical workers, research scientists, volunteers, managers, receptionists and nurses.

*Funding*

The **Millennium Cohort Study**, **1970 British Cohort Study** and **1958 National Child Development Study** are supported by the Centre for Longitudinal Studies, Resource Centre 2015-20 grant (ES/M001660/1) and a host of other co-funders.

**ALSPAC:** The UK Medical Research Council and Wellcome (Grant ref: 217065/Z/19/Z) and the University of Bristol provide core support for ALSPAC. This publication is the work of the authors and E. McElroy and G.P. Ploubidis will serve as guarantors for the contents of this paper. A comprehensive list of grants funding is available on the ALSPAC website (<http://www.bristol.ac.uk/alspac/external/documents/grant-acknowledgements.pdf>).

This research was specifically funded as part of a collaborative research programme entitled ’Cohorts and Longitudinal Studies Enhancement Resources’ (CLOSER) funded by the ESRC (http://www.esrc.ac.uk) (ES/K000357/1).

### Appendix S2.

*Measurement invariance using multigroup confirmatory factor analysis*

We tested the measurement invariance of our harmonised pools of items by fitting a series of nested confirmatory factor models (CFAs), in which increasingly strict equality constraints were placed on specific measurement parameters across the different cohorts. First we tested a configural model in which we estimated correlated emotional and behavioural factors in each of the four cohorts. Model fit was assessed using the chi-square statistic, the comparative fit index (CFI) (Bentler, 1990), and Root Mean Square Error of Approximation (RMSEA) (Steiger, 1990), with CFI values of greater than 0.90 and RMSEA values of less than 0.08 indicating acceptable fit (Hooper, Coughlan, & Mullen, 2008). Next, we tested a scalar invariance model in which the factor loadings and thresholds were held equal across cohort groups. This was then compared with the configural model, by examining differences in fit statistics. Based on established guidelines, values of ∆RMSEA < 0.015 and ∆CFI < 0.01 were judged to support scalar invariance (Chen, 2007). If scalar invariance is supported, this indicates that there are no systematic differences in measurement error in the outcomes of interest that can be attributed to cohort membership (Armstrong, 1998), thus valid comparisons of both means and covariances can be made across studies. We also report the *X*^2^ test between models, although note that is well-documented that this test is unlikely to support invariance when sample sizes are large (Bentler, & Bonett, 1980).

Our tests of measurement invariance were estimated with the weighted least squares, mean and variance adjusted (WLSMV) estimator, and were estimated using Mplus version 8.3 (Muthén & Muthén, 2017). For more in-depth discussions of measurement invariance, we refer readers elsewhere (Putnick & Bornstein, 2016).

### Appendix S3.

*Multiple imputation strategy*

To account for differences in attrition across the four cohorts, estimates of latent means were produced for the best-fitting partial scalar model using multiple imputation, and these values were compared with the estimates produced using or analysed samples. All study variables were included in the imputation models, and imputation was conducted separately by cohort to preserve any differences in mean and covariance structures (Enders & Gottschall, 2011). Research suggests that socio-economic position is a key predictor of attrition in the British cohorts (Mostafa et al., 2021), and the inclusion of our SEP variables in the imputation models is therefore a strength of our imputation approach, given that >98% of the sample had at least one indicator of SEP (see Table S2 for missing data patterns). Twenty imputed data sets were produced using Stata v 17.0 and these were pooled to produce estimates of latent mean differences in Mplus V 8.3 (Muthén & Muthén, 2017).

For the additional analyses in the study (cohort stratified analyses, mediation analysis), missing data was handled using FIML due to the low levels of missingness on our predictor variables.


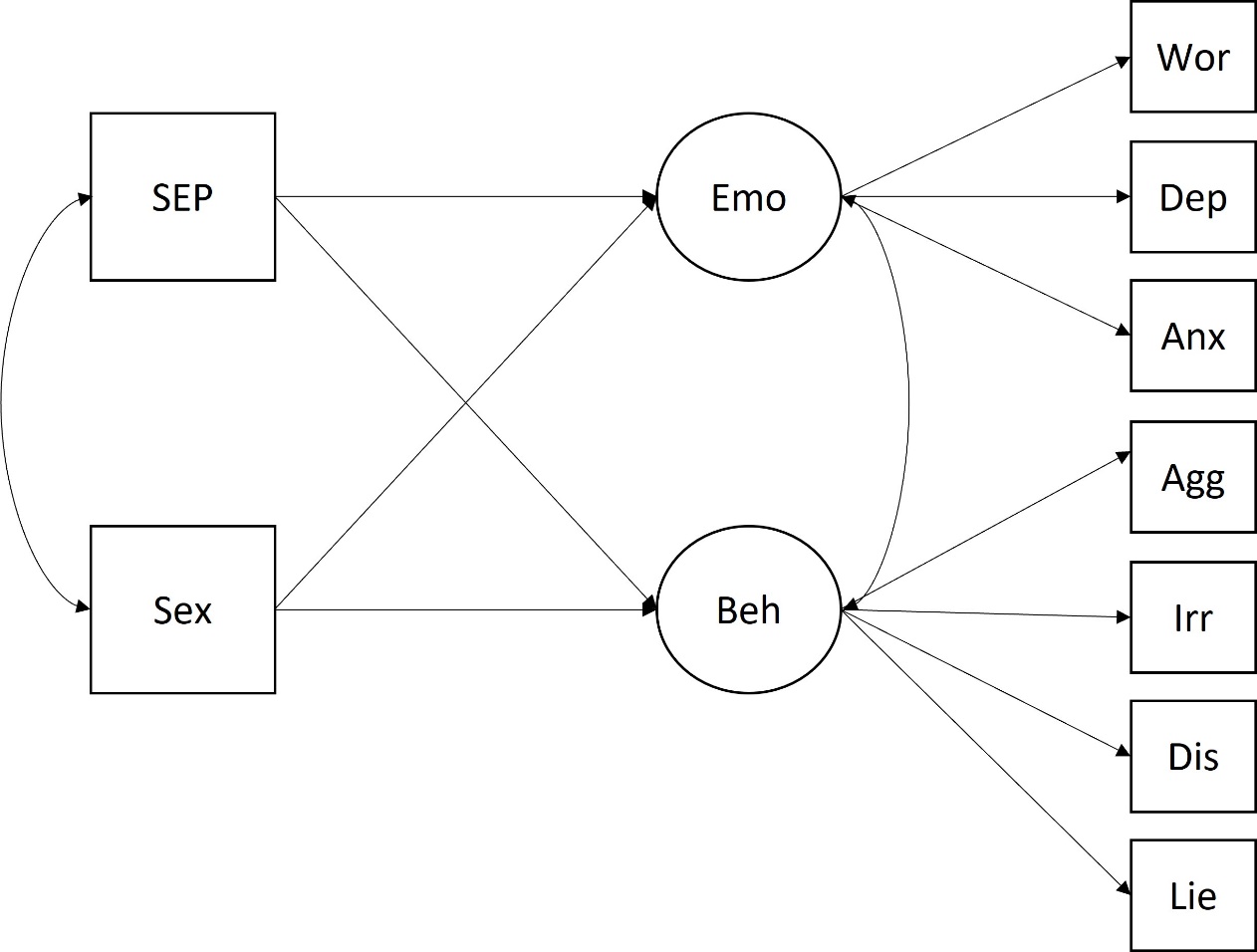


### Figure S1. Diagram of MIMIC models.

Note. SEP indicators (housing tenure, mother’s education, father’s education, father’s occupation) were included as exposures in separate models (4 models x 4 cohorts = 16 separate models). Estimates of the effect of sex on mental health presented in the manuscript (Figures 1-2) come from sex-only models that did not contain SEP exposures. Emo = emotional problems; Beh = behavioural problems; Wor = worries; Dep = depression; Anx = anxiety; Agg= aggression; Irr=irritability; Dis = disobedience; Lie = lies.


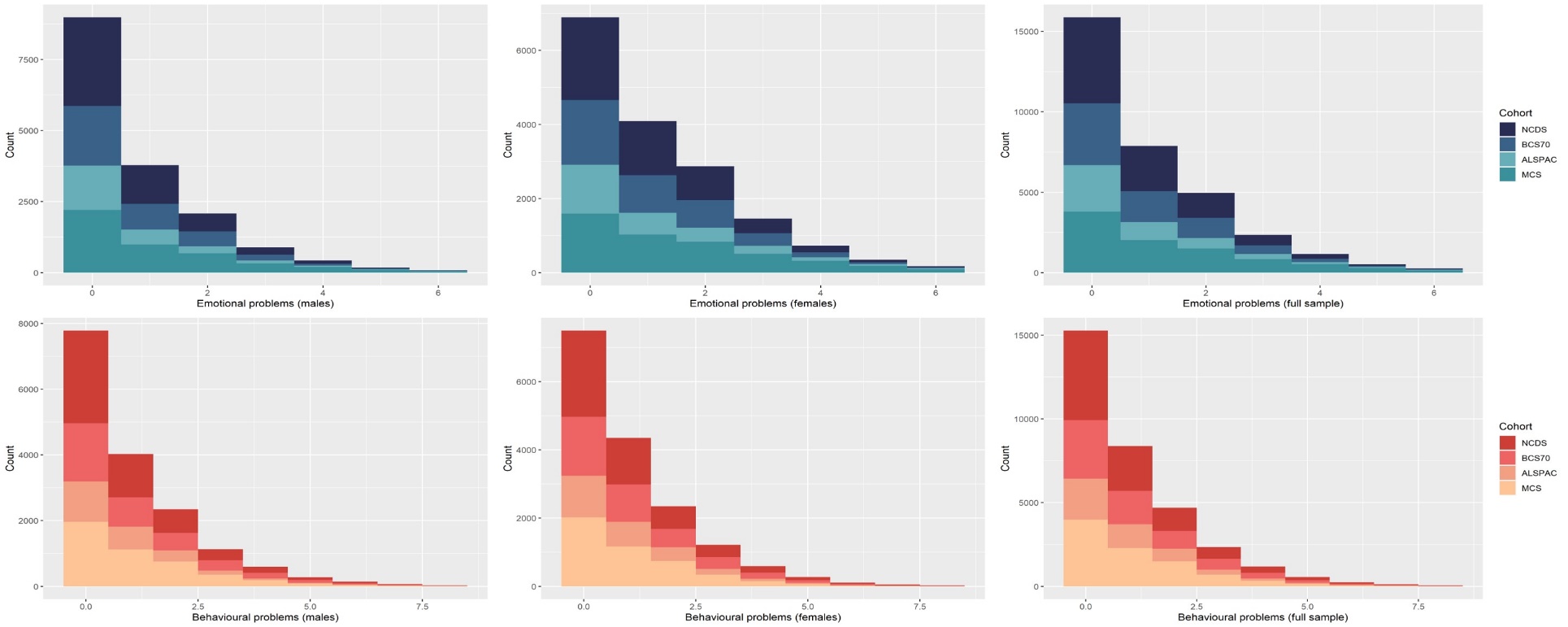


### Figure S2. Distributions of raw score of harmonised emotional and behavioural problem scales (by sex and total sample).


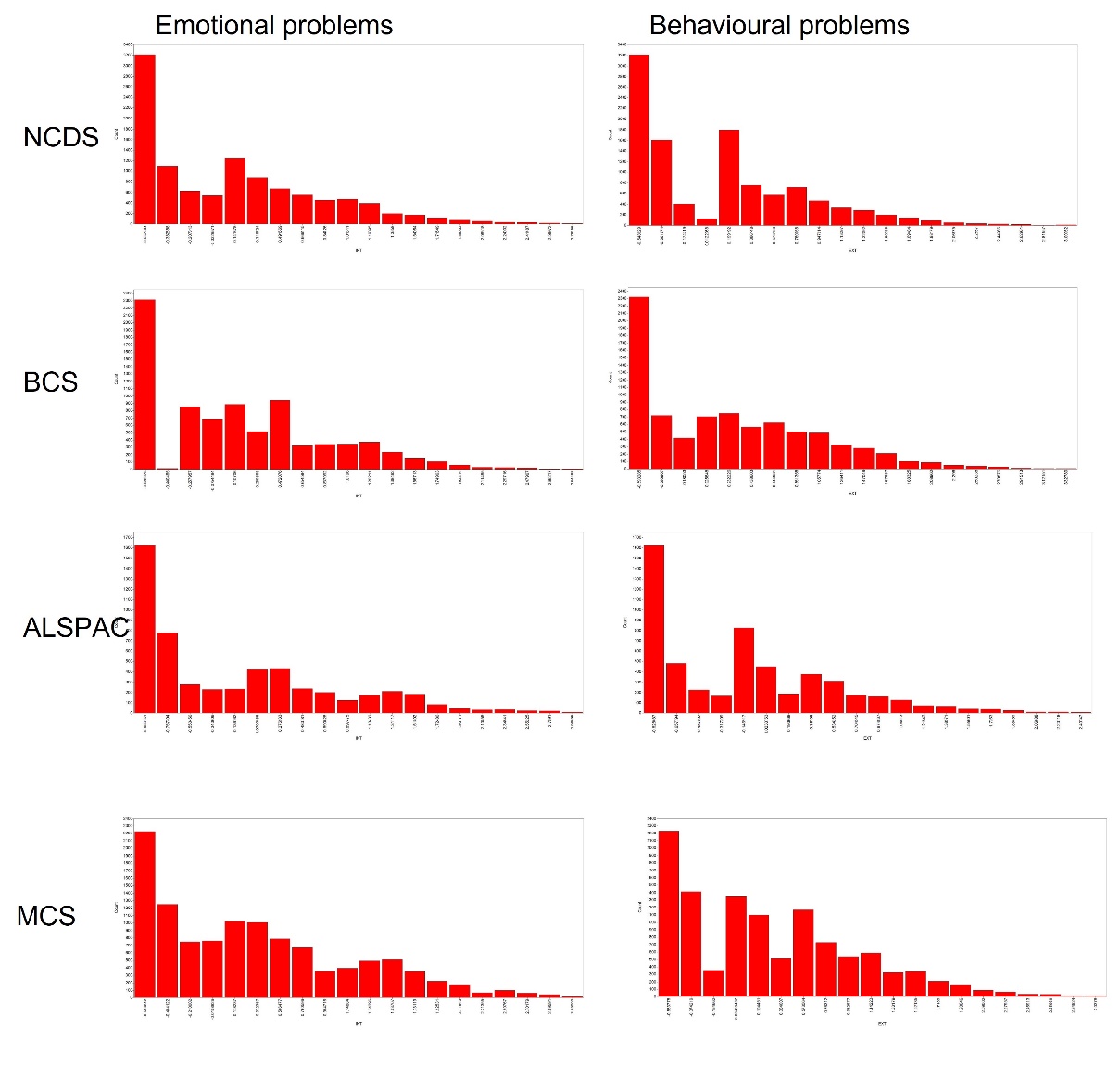


### Figure S3. Latent score distributions of harmonised emotional and behavioural problem scales.


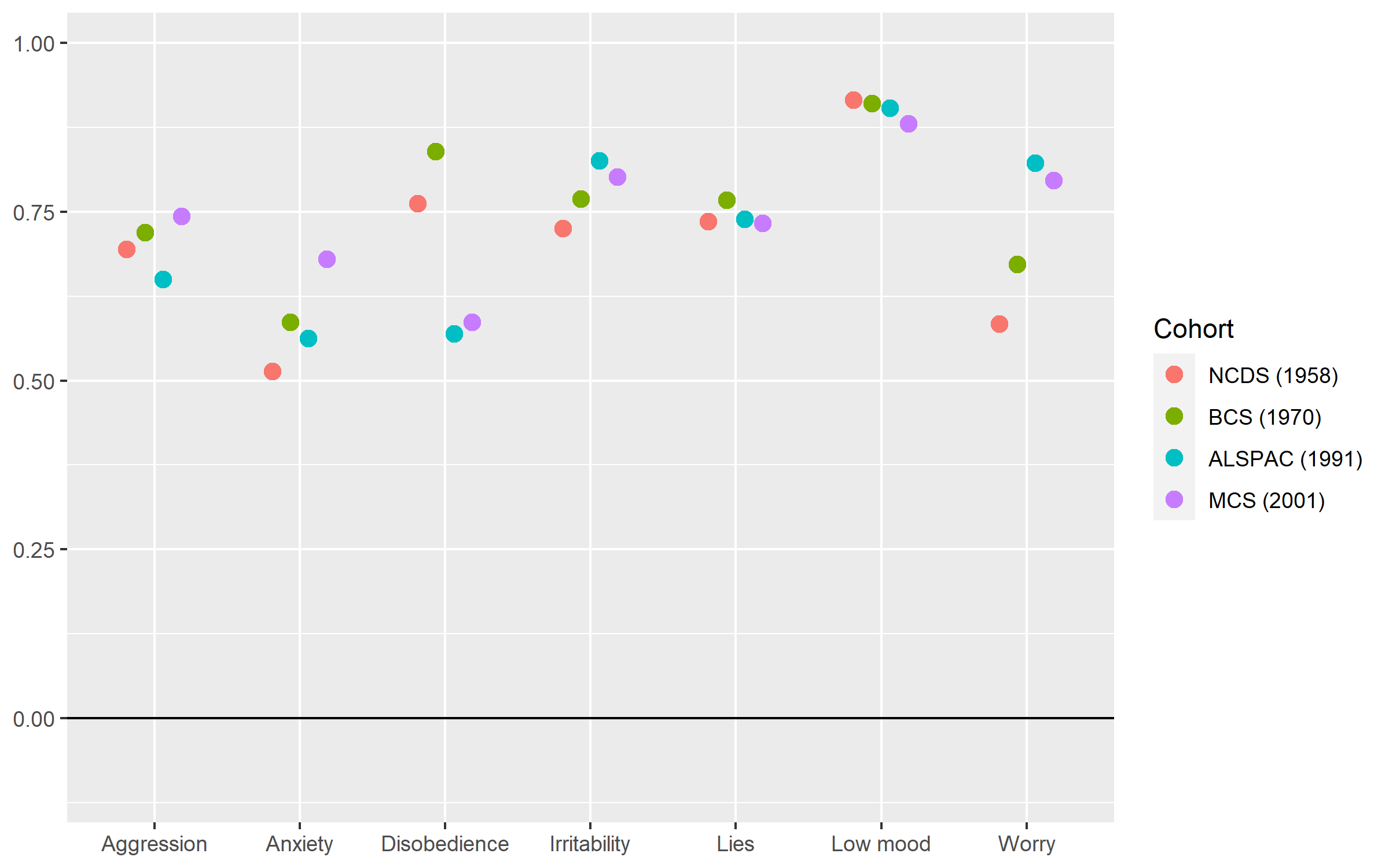


### Figure S4. Standardised factor loadings from configural model (analytic samples).


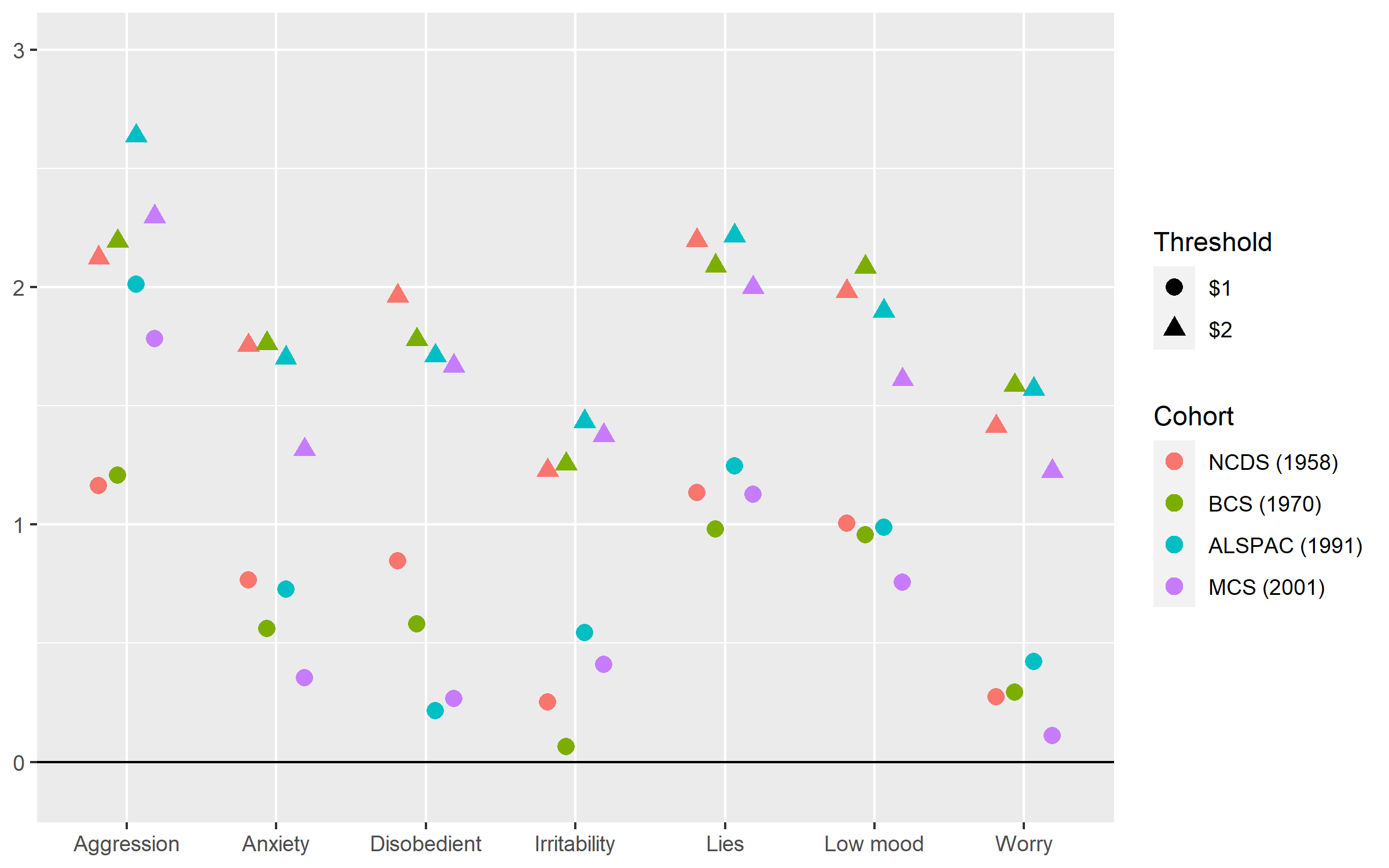


### Figure S5. Standardised thresholds from configural model (analytic samples). Thresholds are on probit scale.


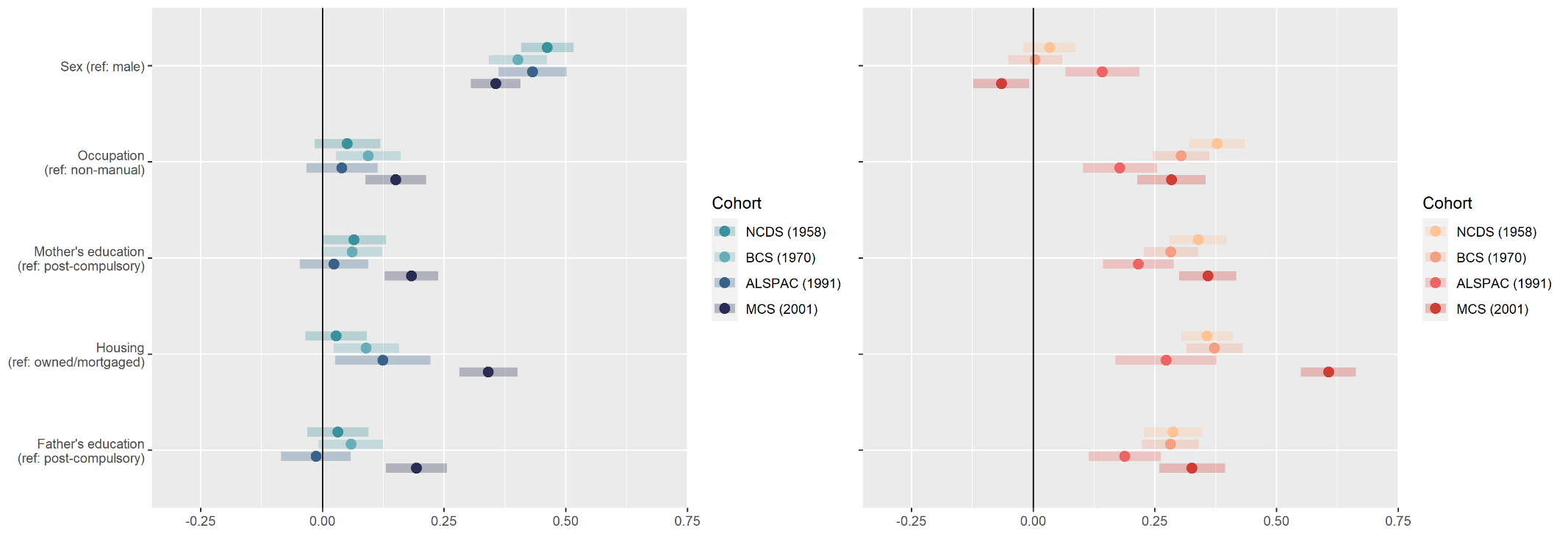


### Figure S6. Sensitivity analysis – cohort stratified analyses with MCS emotional (left) and behavioural (right) problems at age 14/15.

### References

Armstrong, B. G. (1998). Effect of measurement error on epidemiological studies of environmental and occupational exposures. *Occupational and environmental medicine, 55*(10), 651-656.

Bentler, P. M. (1990). Comparative fit indexes in structural models. *Psychological bulletin, 107*(2), 238.

Bentler, P. M., & Bonett, D. G. (1980). Significance tests and goodness of fit in the analysis of covariance structures. Psychological Bulletin, 88(3), 588–606.

Chen, F. F. (2007). Sensitivity of goodness of fit indexes to lack of measurement invariance. *Structural Equation Modeling: A Multidisciplinary Journal, 14*(3), 464-504.

Enders, C. K., & Gottschall, A. C. (2011). Multiple imputation strategies for multiple group structural equation models. *Structural Equation Modeling, 18*(1), 35-54.

Hooper, D., Coughlan, J., & Mullen, M. R. (2008). Structural equation modelling: Guidelines for determining model fit. *Electronic journal of business research methods, 6*(1), 53-60.

Mostafa, T., Narayanan, M., Pongiglione, B., Dodgeon, B., Goodman, A., Silverwood, R. J., & Ploubidis, G. B. (2021). Missing at random assumption made more plausible: evidence from the 1958 British birth cohort. *Journal of Clinical Epidemiology, 136*, 44-54.

Muthén, L. K., & Muthén, B. O. (2017). *Mplus User's Guide. Eighth Edition.* . Los Angeles, CA: Muthén & Muthén.

Putnick, D. L., & Bornstein, M. H. (2016). Measurement invariance conventions and reporting: The state of the art and future directions for psychological research. *Developmental review, 41*, 71-90.

Steiger, J. H. (1990). Structural model evaluation and modification: An interval estimation approach. *Multivariate behavioral research, 25*(2), 173-180.
